# 1869. Knowledge about and Perceptions of Latent Tuberculosis Infection (LTBI) among Physicians and Advanced Practice Providers at Tufts Medical Center

**DOI:** 10.1093/ofid/ofad500.1697

**Published:** 2023-11-27

**Authors:** Cassidy Boomsma, Tine Vindenes

**Affiliations:** Medstar Georgetown University Hospital, Washington, District of Columbia; Tufts Medical Center, Boston, Massachusetts

## Abstract

**Background:**

*Mycobacterium Tuberculosis* (TB) is a leading cause of infectious disease mortality worldwide. Most active TB cases result from reactivation of latent tuberculosis infection (LTBI). A large reservoir of latent TB is a major public health problem worldwide and in the United States. A better understanding of provider perspectives on LTBI could improve a sub-optimal LTBI care cascade.

**Methods:**

A 13-question survey was fielded to attending physicians, fellows, and advanced practice providers working in the infectious disease, pulmonary/critical care, and general medicine departments at Tufts Medical Center (TMC), an academic tertiary care center in Boston, Massachusetts.

**Results:**

The response rate of the survey was 33% (62/186). Only 17 (30.9%) providers were able to correctly identify all indications for screening. When asked how they decide who to treat, 26.8% (15/56) of providers selected that they would refer their patient to another specialist. Overall, provider confidence in LTBI management decreased along the care cascade (Figure 1). Infectious disease providers expressed more confidence in management than general medicine and pulmonary/critical care providers. The two most observed issues/barriers to care for patients were language, reported by 39 (76.5%) providers, and lack of knowledge or understanding about TB, reported by 39 (76.5%) providers. The two most selected interventions to improve LTBI care at TMC were use of in-person language interpreters, chosen by 37 (71.2%) providers, and information pamphlets available in patients’ preferred languages, chosen by 36 (69.2%) providers.

**Figure:**
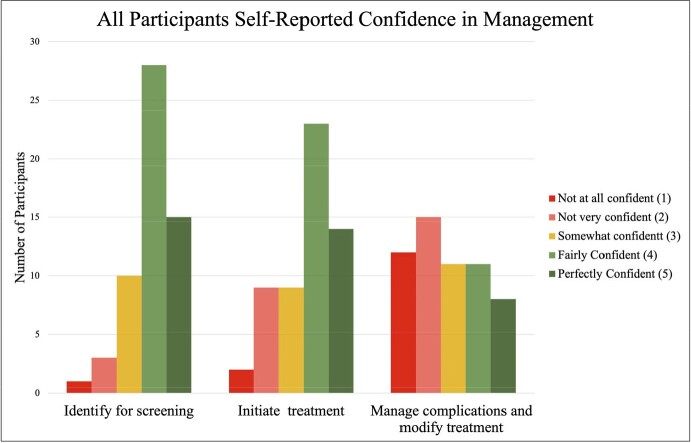

Providers were asked “On a scale of 1-5, (“1” = not at all, and “5” = perfectly) “How confident are you in your ability to do the following” for the listed management tasks.

**Conclusion:**

Results suggest that providers believe the largest barriers to patient LTBI treatment completion are due to a lack of patient comprehension about their infection. Improving patient understanding could be accomplished through in-person interpreters, information pamphlets about LTBI in patients’ preferred languages, and forming community partnerships to improve awareness and understanding of the risks of LTBI. Additionally, ensuring that providers feel confident in their ability to manage LTBI, are updated on screening guidelines, and have referral resources when needed, could help close the gaps in the LTBI care cascade.

**Disclosures:**

**All Authors**: No reported disclosures

